# Quality of Life in HIV Clinical Trials: Why Sexual Health Must Not Be Ignored

**DOI:** 10.1371/journal.pctr.0020008

**Published:** 2007-03-02

**Authors:** Olivier Koole, Christiana Noestlinger, Robert Colebunders

## Sexual Health and Antiretroviral Therapy

Currently more than 20 antiretrovirals are commercially available for treatment of HIV infection [1]. Using them in combination therapy, we are able to treat HIV-infected patients with highly potent regimens and suppress the virus below detectable levels. Correct adherence to these regimens is important to ensure that the viral load will remain undetectable and that the disease does not progress. Moreover, even if adherence is not perfect and the virus becomes resistant to a first-line regimen, there are other treatment options to suppress the virus again to undetectable levels.

Given the choice of several different potent antiretroviral regimens, doctors and patients will tend to prefer those regimens that are easy to take and that have limited short-term and long-term side effects.

One potential side effect of antiretroviral treatment that has received very little scientific attention so far is sexual dysfunction. Sexual dysfunction is defined as difficulty during any stage of the sexual act, including desire, arousal, orgasm, and resolution, that prevents the individual or couple from enjoying sexual activity [2], and, according to DSM-IV (the Diagnostic and Statistical Manual of Mental Disorders, fourth edition) criteria, causing “marked level of distress or interpersonal difficulty” [3]. Sexual dysfunction disorders are generally classified into four categories: sexual desire disorders, sexual arousal disorders, orgasm disorders, and sexual pain disorders [2,3].

Most data relating to the association between antiretroviral therapy and sexual dysfunction are based on cross-sectional studies or case series, and have emerged from industrialised countries [4–12]. Some studies have suggested that antiretrovirals, in particular certain protease inhibitors, may cause sexual problems or dysfunction [4–10]; however, other studies have not confirmed this observation [11,12].

Furthermore, sexual dysfunctions in people infected with HIV may be caused by many different factors. Both organic and psychological factors have been identified, including coping with HIV, pre-existing sexual dysfunctions, sex hormone abnormalities, neuropathy from HIV itself (or related treatment for HIV-caused illnesses), and other iatrogenic causes [13].

Sexual dysfunctions are conceptualised as one component of sexual health, which is an essential element of overall health-related quality of life (HRQOL). While it may include a variety of diverse issues, comprehensive definitions such as the World Health Organization's (WHO) working definition define sexual health as a state of physical, emotional, mental, and social well-being, and not merely the absence of disease, dysfunction, or infirmity. Sexual health encompasses the possibility of having pleasurable and safe sexual experiences [14]. The essence of such lengthy definitions has been summarised in one single statement as “the enjoyment of sexual activity of one's choice, without causing or suffering physical or mental harm” [15]. The latter may pose a particular challenge for people living with HIV/AIDS (PLHA) due to HIV-related stigma and the psychological impact of HIV. As such, sexual health is clearly linked with perceived overall quality of life.

Ideally, we should assess sexual health, including sexual functioning, during randomised clinical trials (RCTs) comparing different antiretroviral regimens, early versus late initiation of antiretroviral therapy, and treatment interruption studies. This could be done by including questions on sexual health in quality of life questionnaires that are administered during such clinical trials. However, quality of life questionnaires that are currently used in HIV clinical trials usually do not include such questions.

## Questionnaires Used in HIV Clinical Trials

The quality of life questionnaires used most widely in HIV clinical trials are the MOS-HIV questionnaire [16] and to a lesser extent the EuroQoL (EQ-5D) questionnaire [17] (Table 1).

**Table 1:**
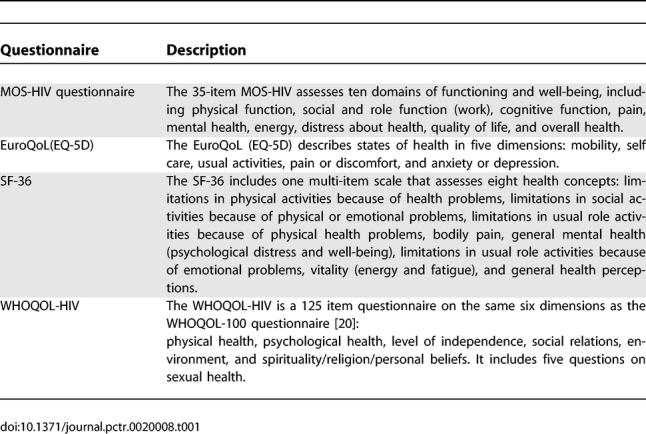
Overview of the Most Commonly Used Questionnaires in Clinical HIV Trials

The MOS-HIV is a brief (generally taking less than ten minutes) comprehensive health status measure based on questions from the Medical Outcomes Study, a large multi-site study of the effects of different ways of delivering medical care [21]. The MOS-HIV was one of the first disease-targeted quality of life questionnaires available for PLHA, and is widely used in clinical trials and other research and evaluation studies. The EQ-5D is a simple instrument assessing only five dimensions of health. In both questionnaires, MOS-HIV and EQ-5D, there are no questions concerning sexual health.

The most widely used health status instrument in other domains is the SF-36 [18] (which is also derived from the Medical Outcomes Study). Other questionnaires assessing quality of life are the quality of well-being scale (QWBS) [22] and the Health Utilities Index (HUI) questionnaire [23]. None of these questionnaires include sexual health questions.

The only questionnaire that does include sexual health questions is the WHO QOL-HIV questionnaire [19]. This questionnaire is based on the WHOQOL-100 questionnaire [20]. Among 120 questions included in this questionnaire, five questions address sexual health and one specifically sexual dysfunction: “Are you bothered by any difficulties in your sex life?” (Box 1).

One hundred and twenty questions pose quite a heavy burden on both the interviewer and the patient/interviewee. In addition, given the sensitive character of questions on sexual health, one may anticipate that these questions would be the first ones to be omitted from a questionnaire during an assessment.

## Why Should Sexual Health Be Assessed During HIV Clinical Trials?

**Figure 1:**
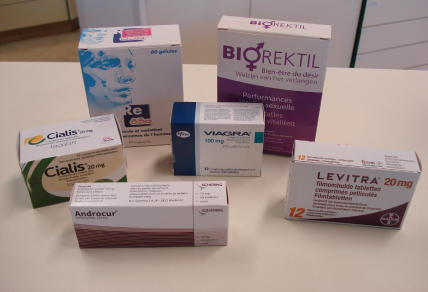
Medications for Sexual Dysfunction Available in One Private Pharmacy in Antwerp, Belgium (Photo: O. Koole)

The importance of sex in most people's lives is illustrated by the fact that there is a growing market for drugs that increase sexual pleasure. The pharmaceutical industry has recently discovered this market, which has resulted in the development of popular erectile dysfunction medications such as sildenafil (Viagra), tadalafil (Cialis), and vardenafil (Levitra) (Figure 1). Evidence from studies in the United Kingdom and United States [24–26] show that these drugs are also commonly used among HIV-infected people, particularly by men who have sex with men (MSM).

A recent study done in the US reported that 11% of MSM used Viagra in the 12 months preceding the interview [26]. Only a proportion of these medications are used for erectile dysfunction; recreational use of these drugs to improve sexual performance is also common.

The annual revenues generated by sales of Viagra and Cialis were respectively over $1 billion and $747 million in 2005 [27,28]. Pharmaceutical companies even have been accused of inventing new diseases, such as sexual dysfunction in women, in order to create new drug markets [29,30]. Other drugs that are widely used to improve libido or sexual pleasure are often unapproved indications of approved drugs (e.g., testosterone and amyl nitrate or “poppers”) or restricted (i.e., illegal) drugs (e.g., crystal methamphetamine) [26,31].

**Box 1. Questions on Sexual Health in WHOQOL-HIV Questionnaire**

1. How well are your sexual needs fulfilled?
2. Are you bothered by any difficulties in your sex life?
3. How satisfied are you with your sex life?
4. How would you rate your sex life?
5. How important to you is your sexual life?

This demonstrates a clear discrepancy between the widespread medicalisation of sexual behaviour and the lack of research in this field. A systematic search of 9,979 abstracts of the 2006 international AIDS conference in Toronto using the keyword “libido” resulted in four abstracts, while searching for the term “sexual dysfunction” resulted in one abstract. However, a search for the keyword “resistance” produced 486 abstracts.

We believe that sexual dysfunction needs more attention from researchers developing clinical trials, since it seems to be quite important from the patients' perspective. Focus group discussions among PLHA performed in Europe have shown that sexual well-being in general was felt to be a crucial part of overall HRQOL, and that sexual dysfunction was perceived to decrease it significantly [32]. PLHA are confronted with many factors that may interfere with their sexual well-being: the psychological impact of the HIV infection itself, the stigma associated with the infection, hormonal abnormalities, fear of transmitting the infection to others, depression, illnesses, and the side effects of drugs such as antiretrovirals. With respect to the latter, PLHA often attribute sexual problems specifically to prescribed drugs which they are taking, and this may ultimately compromise both adherence and secondary prevention. In one study in Italy, sexual dysfunction was associated with poorer adherence to protease inhibitor–containing antiretroviral treatment regimens [33].

Moreover, many drugs have been shown to potentially interfere with sexual functioning (e.g., anti-hypertensive medication, anti-depressive medication, etc.) [34]; thus sexual health questions should be included in all QOL questionnaires during trials with new drugs.

## Conclusion

Sexual health is an important contributing factor to overall quality of life; therefore we propose that specific questions regarding sexual health, including sexual functioning, should be included in quality of life questionnaires in HIV clinical trials.

Moreover, a specific validated questionnaire needs to be developed for studying HIV-related sexual health problems, not only for use in industrialised countries but also in countries with limited resources.

Obviously, besides the importance of posing sexual health questions in HRQOL questionnaires, asking patients about their sexual health during consultations remains essential. While discussing such topics in the context of safer sex practices may increase the quality of life for individual patients, from a public health point of view it can be viewed as contributing to primary HIV prevention. 

## Abbreviations

HRQOL: health-related quality of life
MSM: men who have sex with men
PLHA: people living with HIV/AIDS
RCT: randomised clinical trials
WHO: World Health Organization
